# Artificial intelligence guided HRCT assessment predicts the severity of COVID-19 pneumonia based on clinical parameters

**DOI:** 10.1186/s12879-023-08303-y

**Published:** 2023-05-10

**Authors:** Robert Chrzan, Barbara Wizner, Wojciech Sydor, Wiktoria Wojciechowska, Tadeusz Popiela, Monika Bociąga-Jasik, Agnieszka Olszanecka, Magdalena Strach

**Affiliations:** 1 Department of Radiology, Jagiellonian University Medical College, Kopernika 19, Krakow, 31-501 Poland; 2 Department of Internal Medicine and Gerontology, Jagiellonian University Medical College, Krakow, Poland; 3 Department of Rheumatology and Immunology, Jagiellonian University Medical College, Krakow, Poland; 4 1st Department of Cardiology, Interventional Electrocardiology and Arterial Hypertension, Jagiellonian University Medical College, Krakow, Poland; 5 Department of Infectious Diseases, Jagiellonian University Medical College, Krakow, Poland

**Keywords:** Artificial intelligence, HRCT, COVID-19, Oxygen saturation, Inflammatory biomarkers, Transfer to ICU, In-hospital death

## Abstract

**Background:**

The purpose of the study was to compare the results of AI (artificial intelligence) analysis of the extent of pulmonary lesions on HRCT (high resolution computed tomography) images in COVID-19 pneumonia, with clinical data including laboratory markers of inflammation, to verify whether AI HRCT assessment can predict the clinical severity of COVID-19 pneumonia.

**Methods:**

The analyzed group consisted of 388 patients with COVID-19 pneumonia, with automatically analyzed HRCT parameters of volume: AIV (absolute inflammation), AGV (absolute ground glass), ACV (absolute consolidation), PIV (percentage inflammation), PGV (percentage ground glass), PCV (percentage consolidation). Clinical data included: age, sex, admission parameters: respiratory rate, oxygen saturation, CRP (C-reactive protein), IL6 (interleukin 6), IG - immature granulocytes, WBC (white blood count), neutrophil count, lymphocyte count, serum ferritin, LDH (lactate dehydrogenase), NIH (National Institute of Health) severity score; parameters of clinical course: in-hospital death, transfer to the ICU (intensive care unit), length of hospital stay.

**Results:**

The highest correlation coefficients were found for PGV, PIV, with LDH (respectively 0.65, 0.64); PIV, PGV, with oxygen saturation (respectively − 0.53, -0.52); AIV, AGV, with CRP (respectively 0.48, 0.46); AGV, AIV, with ferritin (respectively 0.46, 0.45). Patients with critical pneumonia had significantly lower oxygen saturation, and higher levels of immune-inflammatory biomarkers on admission. The radiological parameters of lung involvement proved to be strong predictors of transfer to the ICU (in particular, PGV ≥ cut-off point 29% with Odds Ratio (OR): 7.53) and in-hospital death (in particular: AIV ≥ cut-off point 831 cm^3^ with OR: 4.31).

**Conclusions:**

Automatic analysis of HRCT images by AI may be a valuable method for predicting the severity of COVID-19 pneumonia. The radiological parameters of lung involvement correlate with laboratory markers of inflammation, and are strong predictors of transfer to the ICU and in-hospital death from COVID-19.

**Trial registration:**

National Center for Research and Development CRACoV-HHS project, contract number SZPITALE-JEDNOIMIENNE/18/2020.

## Background

At the onset of the COVID-19 pandemic, it was suggested to use lung HRCT (high resolution computed tomography), to confirm positive cases, particularly in centers with limited access to PCR (polymerase chain reaction) test and a large number of new cases [1].

However, it quickly turned out that the specificity of the HRCT is insufficient due to the same radiological symptoms in pneumonia of a different etiology, particularly atypical.

Therefore, in current guidelines of radiological societies (American College of Radiology, British Thoracic Imaging Society), HRCT is not recommended as a screening tool nor as a first-line COVID-19 test [2, 3].

PCR (polymerase chain reaction) from the pharyngeal or nasopharyngeal swab remains the gold standard for COVID-19 verification.

However, the above guidelines state that HRCT can be used in cases of complications in confirmed COVID-19 patients. It is estimated that in about 10% of cases, the severity of the disease requires admission to the ICU (intensive care unit) [4].

AI (artificial intelligence) software with automatic detection and assessment of CT or X-ray images can be a very useful tool in daily practice [5, 6].

In patients with COVID-19, the main advantage of AI is the possibility of a rapid assessment of a large number of images [7], which is particularly important for hospitals with insufficient number of radiologists.

The specificity of the identification of COVID-19 by AI as the etiology of detected pneumonia is still limited [7–9]. However, in patients with PCR confirmed COVID-19, such rapid automatic analysis may be a valuable method for objective assessment of the dynamics of pulmonary lesions in the course of treatment [10] and the early prediction of a severe course of the disease [11], which is very important for the optimal treatment strategy.

The purpose of the study was to compare the results of AI analysis of the extent of pulmonary lesions on HRCT images in COVID-19 pneumonia, with clinical data including laboratory markers of inflammation.

## Methods

From 20 January 2021 to 31 May 2021, in 498 patients hospitalized in the Krakow University Hospital due to COVID-19 infection, diagnosed by PCR (polymerase chain reaction) from nasopharyngeal swabs, clinical and laboratory data were prospectively collected as a part of the CRACoV-HHS (CRAcow in CoVid pandemic — Home, Hospital and Staff) project [12].

One of the exclusion criteria was the history of interstitial lung disease.

In 388 patients of this group, chest HRCT (high resolution computed tomography) was performed, due to the clinical indications concerning COVID-19 pneumonia.

The calculated sample size in our study, using Fisher’s formula, assuming the population size of 10,000, the confidence interval ± 5%, confidence level 95%, standard deviation 0.5, was 384.

Scanning was performed by multirow (64 or 80) helical scanners, using slice thickness of 0.625–1.25 mm, tube current-time product 100–350 mAs, voltage 120 kV.

**Fig. 1:**
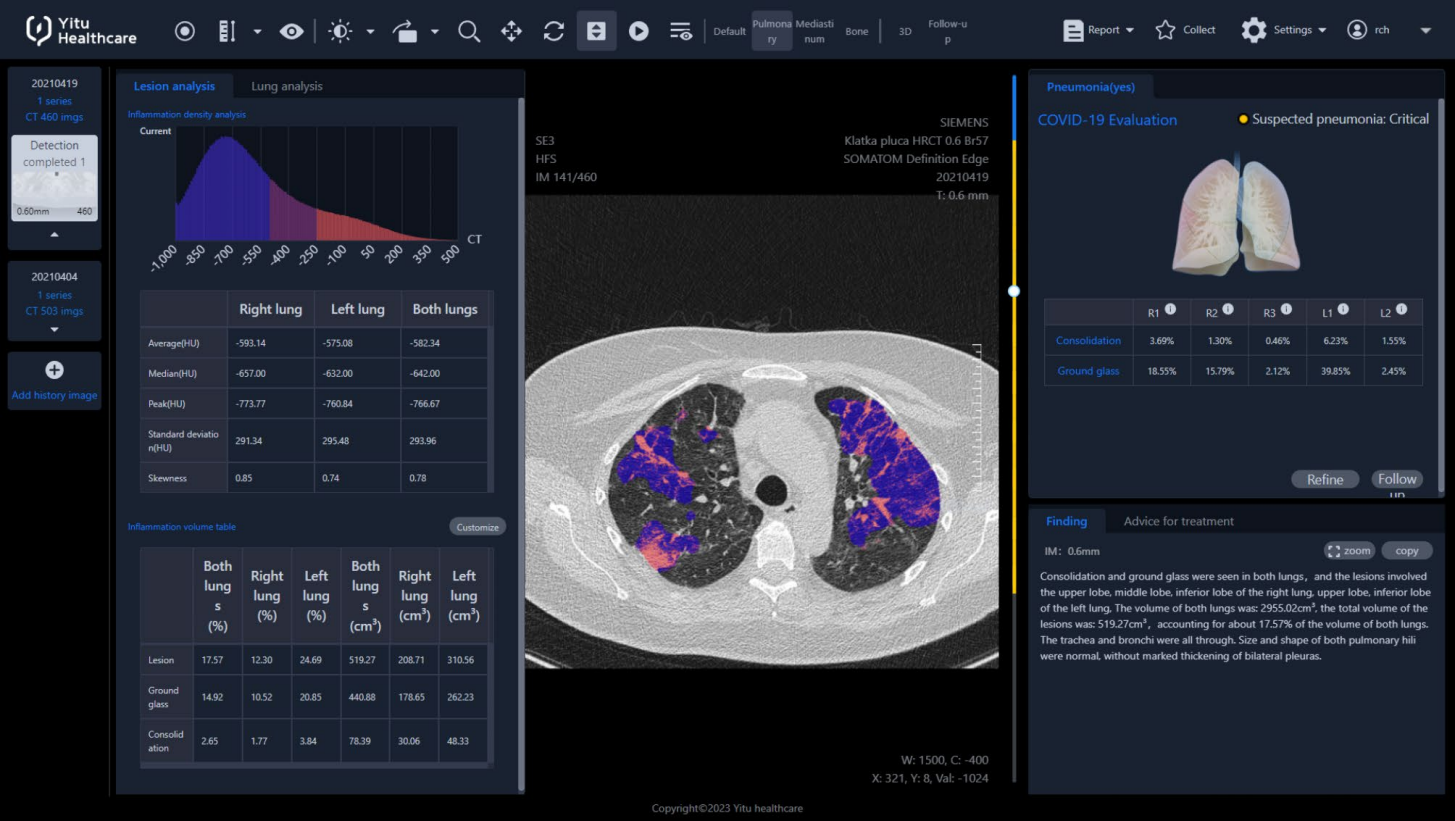
The final report from the automatic analysis of HRCT by AI. Inflammation regions are marked in color depending on attenuation values in Hounsfield units; for example, the areas of ground glass are shown in blue

The analysis of the HRCT images was performed by means of AI technology software created by YITU CT, YITU Healthcare Technology Co., Ltd. and Huawei Technologies Co., Ltd., China [13, 14] (Fig. 1).

The creation and operation of the YITU CT AI is discussed in the publication by Pan et al. [15]. The software consists of three different network components: (1) twelve convolutional segments, including convolutional layer, batch normalization layer, and an activation layer; (2) tree max-pooling layers for down-sampling; and (3) tree transpose convolutional layers for up-sampling. The database of images used to train the AI included chest CT images from 942 confirmed COVID-19 patients and 1340 healthy people, randomly divided into a training set (75%) and a test set (25%). 100 training epochs were performed with a batch size of 8. Adam algorithm was used for the model optimizer. The ground truth region of interest (GT-ROI) for lung lesions was first drawn manually by a radiologist with 5-year experience in thoracic radiology and then corrected if needed and approved by a senior radiologist with 28-year experience in thoracic radiology. The accuracy of the measurement of predicted ROI (PR-ROI) by AI, in reference to GT-ROI, determined by the Dice coefficient, was 85.00% for the training set and 82.08% for the test set.

The YITU CT is commercially available in Asia and Europe, and it has CE certification for its scope. The product meets the provisions of the Council Directive 93/42/EEC on Medical Devices (Class 1, rule 12, medical image management and processing software).

For the purposes of this study, the consecutive radiological parameters were automatically assessed by the software, for both lungs together: AIV - absolute inflammation volume in cm^3^ (inflammation volume = ground glass volume + consolidation volume), AGV - absolute ground glass volume in cm^3^, ACV - absolute consolidation volume in cm^3^, PIV - percentage inflammation volume in relation to the volume of lungs), PGV - percentage ground glass volume in relation to the volume of lungs, PCV - percentage consolidation volume in relation to the volume of lungs, and finally, estimated severity of pneumonia, expressed as none, mild, moderate, or critical.

The clinical data used in this study was retrieved from the CRACoV-HHS database and consisted of age, sex; parameters at admission: respiratory rate, oxygen saturation, CRP (C-reactive protein), IL6 (interleukin 6), IG - immature granulocytes, WBC (white blood count), neutrophil count, lymphocyte count, serum ferritin, LDH (lactate dehydrogenase), NIH (National Institute of Health) severity score; as well as parameters of clinical course: in-hospital death, transfer to the ICU (intensive care unit), and length of hospital stay.

Statistica 13.3 software (TIBCO Software Inc., Palo Alto, CA, USA) was used for statistical calculations.

Initially, we analyzed and compared the radiological and clinical parameters, in the individual subgroups of pneumonia severity, as assessed by AI software, based on the extent of infiltrations on CT images.

Similarly, we analyzed and compared the radiological and clinical parameters, in the subgroups of pneumonia severity, as assessed clinically using the NIH severity score (due to the low number of asymptomatic and critical cases, we used the following groups: asymptomatic or mild, moderate, severe, or critical).

The comparison of the values of categorical variables in the groups was performed using the chi-squared test.

The comparison of the values of continuous variables in the subgroups was performed using the Kruskal–Wallis test. After detecting statistically significant differences, post-hoc analysis with Dunn’s test was performed to identify significantly different groups.

Additionally, we tested the correlation between AI based pneumonia severity and NIH based severity.

Then we assessed the correlations between the radiological parameters of the extent of pneumonia and the clinical parameters on admission.

We used Spearman’s rank correlation coefficient because of non-normal distributions of variables.

In the whole group of patients, we also assessed the usefulness of the radiological parameters of lung involvement for the prediction of transfer to the ICU and in-hospital death from COVID-19, by plotting receiver operating characteristic (ROC) curves, computing the areas under the ROC curves (AUC), and calculating optimal cut-off points using the Youden index.

Finally, we explored the independent predictors of transfer to the ICU and in-hospital death using the multivariable logistic regression analyses. Each model included age, sex, and one of the radiological parameters: AIV, AGV, ACV, PIV, PGV, PCV (≥ vs. < cut-off point as assessed in the ROC curves).

Statistical significance was p < 0.05 in all analyses.

The methodology of this study was similar to that used in our previous work, concerning a different cohort of patients with COVID-19, assessed fully retrospectively [16].

The study was approved by the local bioethics committee (opinions No. 1072.6120.333.2020 dated December 7, 2020, No. 1072.6120.363.2020 dated December 16, 2020).

Each patient signed an informed consent to participate in the proposed medical procedures during the CRACoV-HHS project.

## Results

The final group analyzed consisted of 388 patients: 146 women, 242 men, 23–89 years old, average 60.5 years old, median 63 years old. The median delay between HRCT and hospitalization was 0 days. The median hospitalization time was 12 days. 32 (8.2%) patients were transferred to the intensive care unit (ICU). In-hospital mortality for the whole group was 7.5% (29 of 388 patients).

The comparison of the subgroups of pneumonia severity as assessed by AI software (mild, moderate, and critical) is presented in Table 1. In 8 patients, the intensity of inflammatory changes on CT images was assessed as none by the software, and this subgroup was not included in the table.

**Table 1:** Clinical and radiological parameters in the subgroups of pneumonia severity, as assessed by AI.

Characteristics	AI pneumonia severity group		
	Mild	Moderate	Critical
Number	33	73	274
Age, yr	64.2 (14.4)^C^	65.0 (11.2)^C^	59.1 (12.7)^M,Mo^
Female, n (%)	12 (36.4%)	29 (39.7%)	104 (37.9%)
**Radiological parameters**			
AIV, cm³	45.78 (13.62–94.42)^Mo,C^	333.42 (231.40-540.48)^M,C^	1130.43 (686.28-1708.29)^M,Mo^
AGV, cm³	41.93 (12.75–89.86)^Mo,C^	289.43 (182.16-455.61)^M,C^	879.63 (523.38-1317.99)^M,Mo^
ACV, cm³	2.73 (0.94–9.19)^Mo,C^	36.41 (25.19–73.83)^M,C^	196.19 (123.43-329.54)^M,Mo^
PIV, %	1.00 (0.38–1.91)^Mo,C^	7.30 (5.04–10.53)^M,C^	27.90 (18.28–43.13)^M,Mo^
PGV, %	0.77 (0.37–1.68)^Mo,C^	6.53 (3.91–9.57)^M,C^	22.30 (14.75–35.09)^M,Mo^
PCV, %	0.05 (0.01–0.20)^Mo,C^	0.84 (0.51–1.55)^M,C^	5.28 (3.01–9.51)^M,Mo^
**Parameters on admission**			
Respiratory rate, /min	16 (14–18)	16 (13–18)	16 (15–18)
Oxygen saturation, %	96 (91–97)^C^	94 (89–95)^C^	89 (85–92)^M,Mo^
CRP, mg/l	38.7 (16.9–69.4)^C^	47.1 (22.9–88.9)^C^	82.5 (47.4–139)^M,Mo^
IL6, pg/ml	16.7 (11.2–46.2)	29.5 (12.2–55.9)	39.5 (13.9–69.8)
IG, 10^3/µl	0.02 (0.01–0.05)	0.03 (0.02–0.04)	0.03 (0.02–0.06)
WBC, 10^3/µl	4.92 (4.09–7.52)	5.70 (3.93–6.93)	5.67 (4.43–8.07)
Neutrophil count, 10^3/µl	3.29 (2.42–5.02)	3.84 (2.51–5.84)	4.48 (3.16–6.83)
Lymphocyte count, 10^3/µl	1.01 (0.73–1.35)^C^	0.87 (0.64–1.17)	0.73 (0.58–1.05)^M^
Ferritin, µg/l	498 (235–995)	531 (253–878)^C^	814 (517–1325)^Mo^
LDH, U/l	275 (232–353)^C^	289 (237–359)^C^	386 (316–519)^M,Mo^
NIH score, 0–4	2 (1–2)^C^	2 (2–3)^C^	3 (2–3)^M,Mo^
**Clinical course**			
In hospital death, n (%)	0	7 (9.6%)	22 (8.0%)
Transfer to the ICU, n (%)	0	5 (6.8%)	27 (9.9%)
Length of hospital stay, days	14 (10–21)^Mo^	10 (8–15)^M,C^	12 (9–17)^Mo^

Data shown as mean (SD), median (interquartile range), number (%)

^M^ significantly (p < 0.05) different from Mild group

^Mo^ significantly (p < 0.05) different from Moderate group

^C^ significantly (p < 0.05) different from Critical group

AI – artificial intelligence, AIV - absolute inflammation volume, AGV - absolute ground glass volume, ACV - absolute consolidation volume, PIV - percentage inflammation volume, PGV - percentage ground glass volume, PCV - percentage consolidation volume, CRP - C-reactive protein, IL6 - interleukin 6, IG – immature granulocytes, WBC – white blood cells, LDH – lactate dehydrogenase, NIH score – National Institute of Health severity score, ICU - intensive care unit, SD - standard deviation

The patients with critical pneumonia, as assessed by AI, had significantly lower oxygen saturation, higher levels of CRP and LDH, higher NIH scores, compared to the mild and moderate subgroups, as well as lower lymphocyte count compared to the mild group, and higher level of ferritin, compared to the moderate group.

The comparison of the subgroups of pneumonia severity as assessed clinically using the NIH severity score (asymptomatic or mild, moderate, severe, or critical) is presented in Table 2.

**Table 2:** Clinical and radiological parameters in the subgroups of pneumonia severity, as assessed clinically

Characteristics	NIH severity group		
	Asymptomatic or mild	Moderate	Severe or critical
Number	46	127	215
Age, yr	64.8 (14.4)	59.4 (13.3)	60.2 (12.3)
Female, n (%)	19 (41.3%)	49 (38.6%)	78 (36.3%)
**Radiological parameters**			
AIV, cm³	282.98 (37.44-478.98)^Mo,Se^	568.96 (255.72-1011.46)^As,Se^	1145.98 (620.62-1796.24)^As,Mo^
AGV, cm³	221.92 (33.27–428.60) ^Mo,Se^	477.59 (197.59-812.99) ^As,Se^	893.51 (484.91-1354.22) ^As,Mo^
ACV, cm³	37.45 (4.17–124.80) ^Se^	95.13 (27.75-190.78) ^Se^	191.41 (100.93–348.10) ^As,Mo^
PIV, %	6.83 (0.61–13.53) ^Mo,Se^	14.47 (5.14–24.58) ^As,Se^	29.17 (16.48–45.97) ^As,Mo^
PGV, %	5.97 (0.60-11.03) ^Mo,Se^	11.58 (4.30–21.30) ^As,Se^	22.53 (13.07–37.45) ^As,Mo^
PCV, %	0.97 (0.06–2.52) ^Se^	2.25 (0.66–4.85) ^Se^	5.03 (2.45–9.88) ^As,Mo^
**Parameters on admission**			
Respiratory rate, /min	16 (14–18)	16 (14–18) ^Se^	17 (15–20) ^Mo^
Oxygen saturation, %	95 (93–97) ^Mo,Se^	93 (89–95) ^As,Se^	87 (82–90) ^As,Mo^
CRP, mg/l	29.5 (16.2–63.3) ^Mo,Se^	62.1 (35.3–97.6) ^As,Se^	94.5 (50.9–151.0) ^As,Mo^
IL6, pg/ml	28.4 (9.5–34.8)	31.3 (12.2–62.5)	35.8 (14.2–72.3)
IG, 10^3/µl	0.02 (0.01–0.05) ^Se^	0.03 (0.02–0.05)	0.04 (0.02–0.06) ^As^
WBC, 10^3/µl	5.48 (4.10–7.40)	5.16 (4.15–6.88)	5.92 (4.43–8.36)
Neutrophil count, 10^3/µl	3.60 (2.75–5.24)	3.80 (2.66–5.62) ^Se^	4.61 (3.24–7.23) ^Mo^
Lymphocyte count, 10^3/µl	0.92 (0.65–1.36)	0.82 (0.63–1.14)	0.72 (0.56–1.01)
Ferritin, µg/l	406 (179–679) ^Se^	629 (377–1045) ^Se^	914 (551–1536) ^As,Mo^
LDH, U/l	268 (231–318) ^Mo,Se^	326 (271–401) ^As,Se^	394 (326–524) ^As,Mo^
**Clinical course**			
In hospital death, n (%)	3 (6.5%)	4 (3.1%) ^Se^	22 (10.2%) ^Mo^
Transfer to the ICU, n (%)	1 (2.2%) ^Se^	5 (3.9%) ^Se^	26 (12.1%) ^As, Mo^
Length of hospital stay, days	13 (10–20) ^Mo^	10 (8–14) ^As,Se^	13 (9–18) ^Mo^

Data shown as mean (SD), median (interquartile range), number (%)

^As^ significantly (p < 0.05) different from Asymptomatic or mild group

^Mo^ significantly (p < 0.05) different from Moderate group

^Se^ significantly (p < 0.05) different from Severe or critical group

NIH severity – National Institute of Health severity, AIV - absolute inflammation volume, AGV - absolute ground glass volume, ACV - absolute consolidation volume, PIV - percentage inflammation volume, PGV - percentage ground glass volume, PCV - percentage consolidation volume, CRP - C-reactive protein, IL6 - interleukin 6, IG – immature granulocytes, WBC – white blood cells, LDH – lactate dehydrogenase, ICU - intensive care unit, SD - standard deviation

The patients with severe or critical pneumonia, as assessed clinically, had significantly higher values of AIV, AGV, ACV, PIV, PGV, PCV, compared to the asymptomatic or mild and moderate subgroups.

The number of patients classified by pneumonia severity according to AI assessment and to NIH is presented in Table 3. A significant (p < 0.05) correlation was found between the severity of pneumonia based on AI assessment and the severity based on NIH (correlation coefficient 0.36).

**Table 3:** The number of patients depending on the severity of the pneumonia defined by AI assessment and by NIH.

AI pneumonia severity group	NIH severity group				
	Asymptomatic	Mild	Moderate	Severe	Critical
None	0	3	4	1	0
Mild	3	9	14	7	0
Moderate	3	10	33	27	0
Critical	3	15	76	179	1

Table 4 shows the effects of the correlation analysis between the radiological parameters of inflammation and the clinical parameters on admission. The highest correlation coefficients were present for radiological parameters, especially PGV, PIV, AIV, AGV, and LDH (respectively 0.65, 0.64, 0.64, 0.63), PIV, PGV, AIV, AGV, and oxygen saturation (respectively − 0.53, -0.52, -0.50, -0.49), AIV, AGV, PIV, PGV, and CRP (respectively 0.48, 0.46, 0.46, 0.45), AGV, AIV, PGV, PIV, and ferritin (respectively 0.46, 0.45, 0.39, 0.36).

**Table 4:** Correlations of radiological parameters of lung involvement and clinical parameters on admission

Variable	Spearman Rank Order Correlations All correlations are significant at p < 0.05										
	Respiratory rate, /min	Oxygen saturation, %	CRP, mg/l	IL6, pg/ml	IG, 10^3/µl	WBC, 10^3/µl	Neutrophil count, 10^3/µl	Lymphocyte count, 10^3/µl	Ferritin, µg/l	LDH, U/l	NIH score, 0–4
AIV, cm^3^	0.23	-0.50	0.48	0.21	0.45	0.24	0.33	-0.24	0.45	0.64	0.46
AGV, cm^3^	0.21	-0.49	0.46	0.19	0.43	0.23	0.31	-0.23	0.46	0.63	0.44
ACV, cm^3^	0.24	-0.47	0.43	0.23	0.39	0.22	0.30	-0.23	0.31	0.52	0.42
PIV, %	0.24	-0.53	0.46	0.21	0.44	0.25	0.33	-0.24	0.36	0.64	0.48
PGV, %	0.23	-0.52	0.45	0.20	0.44	0.25	0.33	-0.24	0.39	0.65	0.47
PCV, %	0.23	-0.47	0.39	0.22	0.37	0.21	0.28	-0.21	0.22	0.48	0.41

AIV - absolute inflammation volume, AGV - absolute ground glass volume, ACV - absolute consolidation volume, PIV - percentage of inflammation volume, PGV - percentage of ground glass volume, PCV - percentage of consolidation volume, CRP - C-reactive protein, IL6 - interleukin 6, IG - immature granulocytes, WBC – white blood cells, LDH – lactate dehydrogenase, NIH score – National Institute of Health severity score

The highest values of AUC in the prediction of transfer to the ICU were found for PGV (0.76), AIV, AGV, PIV (0.75 for all these parameters) (Table 5).

**Table 5:** Receiver operating characteristic (ROC) curve analyses of the radiological parameters of lung involvement for the prediction of transfer to the ICU.

Radiological parameters	The area under curve (AUC)	95% confidence interval	p	Cut-off point	Sensitivity	Specificity	Youden Index
AIV, cm³	0.75	0.67–0.83	0.0000	1434	0.66	0.80	0.46
AGV, cm³	0.75	0.67–0.83	0.0000	1065	0.69	0.76	0.45
ACV, cm³	0.68	0.60–0.77	0.0000	142	0.78	0.53	0.31
PIV, %	0.75	0.66–0.83	0.0000	29	0.72	0.71	0.42
PGV, %	0.76	0.67–0.84	0.0000	29	0.66	0.80	0.46
PCV, %	0.67	0.58–0.75	0.0003	2.5	0.88	0.43	0.30

AIV - absolute inflammation volume, AGV - absolute ground glass volume, ACV - absolute consolidation volume, PIV – percentage of inflammation volume, PGV - percentage of ground glass volume, PCV - percentage of consolidation volume

The highest values of AUC in the prediction of in-hospital death from COVID-19 were found for AIV, AGV, PIV, and PGV (0.64 for all these parameters) (Table 6).

**Table 6:** Receiver operating characteristic (ROC) curve analyses of the radiological parameters of lung involvement for the prediction of in-hospital death

Radiological parameters	The area under curve (AUC)	95% confidence interval	p	Cut-off point	Sensitivity	Specificity	Youden Index
AIV, cm³	0.64	0.55–0.74	0.0029	831	0.79	0.53	0.32
AGV, cm³	0.64	0.55–0.74	0.0031	520	0.83	0.45	0.28
ACV, cm³	0.61	0.51–0.70	0.0304	142	0.69	0.52	0.20
PIV, %	0.64	0.54–0.74	0.0056	15	0.86	0.40	0.27
PGV, %	0.64	0.55–0.74	0.0033	20	0.66	0.60	0.26
PCV, %	0.59	0.50–0.69	0.0574	2.9	0.72	0.46	0.18

AIV - absolute inflammation volume, AGV - absolute ground glass volume, ACV - absolute consolidation volume, PIV – percentage of inflammation volume, PGV - percentage of ground glass volume, PCV - percentage of consolidation volume.

In multivariable logistic regression analyses, including age, sex, and one of the parameters: AIV, AGV, ACV, PIV, PGV, PCV (≥ vs. < cut-off point from the ROC curve) for each model, the radiological parameters proved to be strong predictors of transfer to the ICU (Table 7). In particular, PGV ≥ cut-off point 29% indicated the risk of transfer to the ICU with Odds Ratio [OR]: 7.53, while taking into account age (≥ median 63 years) OR: 2.25; sex (men) OR: 1.01. Next, AIV ≥ cut-off point 1434 cm^3^ indicated the risk with OR: 6.69, while accounting for age (≥ median 63 years) OR: 2.25; sex (men) OR: 1.01.

**Table 7:** The effects of multivariable logistic regression analyses for the prediction of transfer to the ICU.

Radiological parameters	Odds Ratios (95% confidence limits)	p
AIV, cm³	6.69 (3.12–14.33)	0.0000
AGV, cm³	5.99 (2.78–12.93)	0.0000
ACV, cm³	3.32 (1.45–7.59)	0.0044
PIV, %	5.95 (2.66–13.28)	0.0000
PGV, %	7.53 (3.47–16.33)	0.0000
PCV, %	4.02 (1.51–10.69)	0.0052

Every model includes: age, sex, and one of the radiological parameters: AIV, AGV, ACV, PIV, PGV, PCV (≥ vs. < cut-off point from ROC curve). AIV - absolute inflammation volume, AGV - absolute ground glass volume, ACV - absolute consolidation volume, PIV - percentage inflammation volume, PGV - percentage ground glass volume, PCV - percentage consolidation volume

Similarly, in multivariable logistic regression analyses, including age, sex, and one of the parameters: AIV, AGV, ACV, PIV, PGV, PCV (≥ vs. < cut-off point from the ROC curve) for each model, the radiological parameters proved to be strong predictors of in-hospital death (Table 8). In particular, AIV ≥ cut-off point 831 cm^3^ indicated the risk of in-hospital death with OR: 4.31, while taking into account age (≥ median 63 years) OR: 4.03; sex (men) OR: 0.99. Next, AGV ≥ cut-off point 520 cm^3^ indicated the risk with OR: 3.90, while accounting for age (≥ median 63 years) OR: 4.03; sex (men) OR: 0.99.

**Table 8:** The effects of multivariable logistic regression analyses for the prediction of in-hospital death

Radiological parameters	Odds Ratios (95% confidence limits)	p
AIV, cm³	4.31 (1.71–10.84)	0.0019
AGV, cm³	3.90 (1.46–10.46)	0.0068
ACV, cm³	2.02 (0.91–4.47)	0.0823
PIV, %	3.25 (2.21–8.72)	0.0191
PGV, %	2.16 (1.00–4.67)	0.0489
PCV, %	2,21 (0.95–5.12)	0.0647

Every model includes: age, sex, and one of the radiological parameters: AIV, AGV, ACV, PIV, PGV, PCV (≥ vs. < cut-off point from ROC curve).AIV - absolute inflammation volume, AGV - absolute ground glass volume, ACV - absolute consolidation volume, PIV - percentage inflammation volume, PGV - percentage ground glass volume, PCV - percentage consolidation volume

## Discussion

In our study, we confirmed that the extent of pulmonary lesions in the course of COVID-19 pneumonia, analyzed automatically in HRCT by AI, correlated with the intensity of inflammation assessed in laboratory tests, and was a strong predictor of transfer to the ICU or death during hospitalization.

In previous studies, other authors assessed the correlations between the extent of COVID-19 pulmonary infiltrations, assessed in HRCT by radiologists, and the laboratory markers of inflammation and clinical data.

Wang [17] measured the diameter of the largest lung infiltration on CT images, and assessed CRP on admission in 27 patients with COVID-19. In four subgroups based on the intensity of clinical symptoms, the mean values were, respectively: diameter 1.23, 2.94, 9.15, 17.00 cm, CRP 1.52, 16.76, 54.15, 105.00 mg/l. In our research we also found a positive correlation between the radiological parameters of lung involvement and CRP (r from 0.39 to 0.48), with median CRP in severity subgroups: mild, moderate, and critical, respectively 38.7, 47.1, 82.5 mg/l.

Shen [18] compared the clinical data on admission of 36 patients with COVID-19 to the results of CT performed, scored as for the extent of involvement by two radiologists using the method proposed by Chung et al. [19]. The involvement of each of the five lung lobes was classified as score 0 - none (0%), score 1 - minimal (1–25%), score 2 - mild (26–50%), score 3 - moderate (51–75%), or score 4 - severe (76–100%). The ‘total severity score’ was reached by adding the five lobe scores (range of scores, 0–20) and was positively correlated with neutrophil count (r = 0.385) and negatively correlated with lymphocyte count (r = -0.495). In our study we also found a positive correlation between the radiological parameters of lung involvement and neutrophil count (r from 0.28 to 0.33) and a negative correlation between the radiological parameters of lung involvement and lymphocyte count (r from − 0.21 to -0.24).

Francone [20] assessed COVID-19 pneumonia in 130 patients, using a slightly different CT severity scoring method described by Pan et al. [21]. For each of the five lobes, the score from 0 to 5 (0 no involvement; 1, < 5% involvement; 2, 5–25% involvement; 3, 26–50% involvement; 4, 51–75% involvement; and 5, > 75% involvement) was calculated and finally summed up resulting in a global CT score (0 to 25). In this study, significant correlations were found between CT score and CRP (r = 0.6204) as well as D-dimer (r = 0.6625) levels. A CT score of ≥ 18 was associated with an increased mortality risk and was found to be predictive of death both in the univariate analysis (hazard ratio HR, 8.33; 95% confidence interval CI, 3.19–21.73; p < 0.0001) and in the multivariate analysis (HR, 3.74; 95% CI, 1.10–12.77; p = 0.0348). We found a slightly weaker correlation (r from 0.39 to 0.48) between the radiological parameters of lung involvement and CRP. In our study, the radiological parameters of lung involvement > = established cut-off points also proved to be strong predictors of in-hospital death, in particular AIV ≥ cut-off point 831 cm^3^ - OR: 4.31, AGV ≥ cut-off point 520 cm^3^ - OR: 3.90.

Carubbi [22], in 61 patients with COVID-19, collected clinical data including laboratory tests and pulmonary involvement in HRCT, using two semiquantitative scoring systems. In score A, every lobe was scored on a scale of 0 to 3 (0: no lesion, 1: < 1/3 of the lobe volume involved, 2: > 1/3 and < 2/3 of the lobe volume involved, 3: > 2/3 of the lobe volume involved). In score B, every lobe was scored on a scale of 0 to 4 (0: none 0%, 1: minimal 1 to 25%, 2: mild 26 to 50%, 3: moderate 55 to 75%, 4: severe 76 to 100%). A ‘total severity score’ was reached by summing the five lobe scores (score A range 0–15; score B range 0–20). The highest correlation coefficients were found between CT scores and CRP (score A, 0.532; score B, 0.473), ferritin (score A, r = 0.529; score B, r = 0.548), LDH (score A, 0.518; score B, 0.564). We also observed the highest correlation coefficients between radiological parameters and LDH (0.48–0.65), CRP (0.39–0.48) and ferritin (0.22–0.46). Interestingly, Carrubi found that ferritin levels above the 25th percentile were associated with severe pulmonary involvement in CT, but not with the outcome of the disease.

In our study, the main innovation is the automatic analysis of HRCT images by AI. In such analysis, the assessment of the extent of lung involvement by inflammatory lesions is not a time-consuming manual task performed by radiologists, but it is completed automatically within a few minutes after CT.

Such technology has already been used in several centers, but on smaller groups of patients [10, 23].

Pang [23], using the same YITU Healthcare Technology software, analyzed HRCT images of 140 COVID-19 patients, with the assessment of PIV, PGV, and PCV. He found that PIV with a cutoff value of 22.6%, had the highest performance in predicting critical illness, defined as a composite of admission to the intensive care unit, respiratory failure that required mechanical ventilation, shock, or death (AUC 0.868, sensitivity 81.3%, and specificity 80.6%). PIV had a positive correlation with neutrophil count (r = 0.535), erythrocyte sedimentation rate (r = 0.567), D-dimer (r = 0.444), hsCRP (r = 0.495), aspartate aminotransferase (r = 0.410), LDH (r = 0.644), and urea nitrogen (r = 0.439), while negative correlation with lymphocyte count (r = − 0.535). This is in line with our results.

The results of this study that include prospectively collected patients are also similar to the results of our previous study on a different cohort of patients with COVID-19, evaluated fully retrospectively [16]. For example, in this study, the highest correlation coefficients between radiological parameters and laboratory markers are as follows: LDH (0.48–0.65), CRP (0.39–0.48) and ferritin (0.22–0.46); in the previous study: LDH (0.47–0.52), CRP (0.44–0.48) and ferritin (0.34–0.41). Currently, the highest predictive values of in-hospital death from COVID-19 for AIV, AGV, PIV, and PGV (0.64 for all these parameters) were only slightly lower from the values in the previous study: PCV (AUC 0.69), ACV (AUC 0.68) and PIV (AUC 0.67).

For the prospectively collected patients in this study, AIV ≥ cut-off point 831 cm^3^ indicated the risk of in-hospital death with Odds Ratio [OR]: 4.31, next, AGV ≥ cut-off point 520 cm^3^ indicated the risk with OR: 3.90; for the previously retrospectively assessed patients ACV ≥ cut-off point 246 cm^3^ was associated with higher risk of in-hospital death with OR: 4.08, next.

PCV ≥ cut-off point 8.2% was associated with a higher risk of in-hospital death with OR: 4.05.

Interestingly, in our previous study, the admission rate to the ICU was significantly higher in patients with critical pneumonia (25.4%) compared to those with mild (5.9%) or moderate (8.3%) pneumonia. In the current study, the admission to the ICU in patents with critical pneumonia was much lower (9.9%), with no significant difference with moderate (6.8%) pneumonia.

Similarly, in the previous study, the in-hospital death rate was significantly higher in patients with critical pneumonia (16.5%) compared to those with mild (6.8%) or moderate (9.9%) pneumonia. In the current study the in-hospital death rate in patents with critical pneumonia was much lower (8.0%), with no significant difference with moderate (9.6%) pneumonia.

Such differences may be explained by bias in selection to a prospective study, and by different hospitalization dates for two cohorts: from 20 January 2021 to 31 May 2021 for the current prospective study and from 6 March 2020 to 15 October 2020 for the previous retrospective study. Such a time interval between cohorts may result in a different virus mutation and better treatment options.

Our study has, of course, several weaknesses. The accuracy of the inflammation volume measurement by the AI system used in our study was not verified by a different method (manual segmentation or another AI system). The classification criteria used by the system to define the severity subgroups were not available to the user. Many of the COVID-19 patients may have had concomitant infections of a different etiology, and pulmonary embolism, affecting the extent of pulmonary lesions. The AI system was unable to distinguish the infiltrations caused by COVID-19 from the lesions resulting from coinfections and pulmonary embolism, which is an important limitation of the study.

In our study, most (179 of 274) of the patients assessed as “critical” by AI were classified as “severe” by NIH. It means that the term “critical” used by the AI software creators should be rather regarded as “severe”.

Finally, the analysis of HRCT images by AI may be a valuable method to predict the severity of COVID-19 pneumonia. Radiological parameters of lung involvement correlate with laboratory markers of inflammation, and, especially AIV, AGV, are strong predictors of transfer to the ICU and in-hospital death from COVID-19. Automatic separation into pneumonia severity groups based on CT enables the prediction of the degree of clinical disorders. AI based HRCT reading saves time, allowing early decision of admission area and hence the therapy, which is of great clinical relevance.

## Conclusions

In conclusion, the automatic assessment of HRCT images of patients with COVID-19 pneumonia by AI may be a valuable technique for predicting the clinical severity, allowing quick selection of the optimal therapy. Such analysis can and should become a diagnostic imaging method used in everyday practice.

## Data Availability

The data that support the findings of this study are available from the CRACoV-HHS project at the Krakow University Hospital, but restrictions apply to the availability of these data, which were used under license for the current study, and so are not publicly available. Data are however available from the authors upon reasonable request and with permission of the CRACoV-HHS project management. The corresponding author is the contact person for data availability requests.

## List of abbreviations

ACV: Absolute consolidation
AGV: Absolute ground glass
AI: Artificial intelligence
AIV: Absolute inflammation
AUC: Areas under the ROC curves
CI: Confidence interval
CRP: C-reactive protein
HR: Hazard ratio
HRCT: High resolution computed tomography
ICU: Intensive care unit
IG: Immature granulocytes
IL6: Interleukin 6
LDH: Lactate dehydrogenase
NIH: National Institute of Health
OR: Odds ratio
PCR: Polymerase chain reaction
PCV: Percentage consolidation
PGV: Percentage ground glass
PIV: Percentage inflammation
ROC: Receiver operating characteristic curves
WBC: White blood count
